# The Relationship Between the Serum Aspartate Aminotransferase/Alanine Aminotransferase Ratio and the Occurrence and Progression of Abdominal Aortic Aneurysms: A Cross-Sectional Study

**DOI:** 10.21470/1678-9741-2025-0157

**Published:** 2026-02-18

**Authors:** Hande İştar, Bugra Harmandar, Melike Korkmaz Toker, Gokhan Ilhan, Kadir Arslan, Muruvvet Funda Tetik Saruhan

**Affiliations:** 1 Department of Cardiovascular Surgery, Faculty of Medicine, Muğla Sıtkı Kocman University, Mugla, Türkiye; 2 Department of Anaesthesiology and Reanimation, Faculty of Medicine, Muğla Sıtkı Kocman University, Mugla, Türkiye; 3 Department of Cardiovascular Surgery, Mugla Research and Training Hospital, Mugla, Türkiye

**Keywords:** Abdominal Aortic Aneurysm, Biomarkers, Inflammation, Hyperlipidemia, Risk Stratification.

## Abstract

**Introduction:**

This study investigated the relationship between the serum aspartate aminotransferase to alanine aminotransferase (AST/ALT) ratio, the presence and progression of abdominal aortic aneurysms (AAA), assessing its potential as an accessible biochemical marker for patients at risk of rapid aneurysmal growth.

**Methods:**

A total of 180 patients were retrospectively analyzed: 90 with AAA and 90 ageand risk factor-matched controls. Demographic characteristics, risk factors, laboratory parameters, and imaging data were reviewed. The AAA group was divided into rapid and slow enlargement subgroups based on six-month computed tomography measurements. Logistic regression, receiver operating characteristic (ROC) analyses were used to evaluate predictive and discriminative performance, and quartile analysis explored potential threshold effects.

**Results:**

AST/ALT ratio, triglycerides, low-density lipoprotein (LDL) cholesterol, and white blood cell (WBC) count were significantly higher in patients with AAA. Rapid AAA enlargement group had higher AST/ALT ratios, triglycerides, LDL cholesterol, and WBC counts. The AST/ALT ratio was independently associated with AAA presence (odds ratio 2.63; 95% confidence interval [CI] 1.44 - 5.09; P = 0.002) but not with rapid progression (P = 0.10). ROC analysis showed good discrimination for AAA presence (area under the curve [AUC] = 0.72; 95% CI 0.65 - 0.79) and moderate ability for rapid enlargement (AUC = 0.65; 95% CI 0.54 - 0.76). Quartile-based analysis revealed a stepwise increase in AAA prevalence with higher AST/ALT categories.

**Conclusion:**

An elevated AST/ALT ratio is associated with AAA presence but does not independently predict progression, suggesting it reflects hepatic-vascular inflammatory interaction rather than serving as a prognostic marker.

## INTRODUCTION

**Table:** 

Abbreviations, Acronyms & Symbols				
AAA	= Abdominal aortic aneurysms		HDL	= High-density lipoprotein
ALT	= Alanine aminotransferase		IL	= Interleukin
AST	= Aspartate aminotransferase		LDL	= Low-density lipoprotein
AUC	= Area under the curve		MCH	= Mean cell haemoglobin
CAD	= Coronary artery disease		MMP	= Matrix metalloproteinase
CI	= Confidence interval		OR	= Odds ratio
CT	= Computed tomography		ROC	= Receiver operating characteristic
ECM	= Extracellular matrix		STAT3	= Signal transducer and activator of transcription-3
FIB-5	= Fibrosis-5		VIF	= Variance inflation factors
GP130	= Glycoprotein-130		WBC	= White blood cell
HB	= Haemoglobin			

Abdominal aortic aneurysm (AAA) is a progressive vascular condition predominantly affecting the elderly population. Its pathogenesis is multifactorial, involving inflammation, oxidative stress, extracellular matrix (ECM) degradation, and vascular smooth muscle cell apoptosis^[1]^. While traditional risk factors such as smoking, hypertension, and dyslipidaemia have been well established, recent attention has turned toward biochemical markers that might offer insights into early diagnosis.

Among these, the aspartate aminotransferase (AST) to alanine aminotransferase (ALT) ratio - commonly referred to as the De Ritis ratio - has been historically used as a surrogate marker for hepatic fibrosis^[2]^. However, recent studies suggest that this ratio may also be indicative of systemic metabolic stress and vascular dysfunction. For example, Liu et al.^[3]^ demonstrated a significant association between elevated AST/ALT ratios and the presence of peripheral artery disease in a hypertensive population.

The ratio of AST to ALT - historically used as a serological indicator of hepatic fibrosis - has also been linked to cardiovascular and vascular pathologies. Recent studies have demonstrated its association with atherosclerotic burden, arterial stiffness, and peripheral artery disease, suggesting that elevated AST/ALT ratios may reflect systemic metabolic stress and endothelial dysfunction^[4-6]^. Despite these emerging vascular associations, no previous research has directly examined whether this ratio relates to the progression of AAA.

To the best of our knowledge, this is the first study to directly evaluate the association between the serum AST/ALT ratio and the progression of AAA, rather than its mere presence. Previous reports have explored transaminase ratios in cardiovascular and metabolic diseases, but none have specifically linked hepatic enzyme imbalance to aneurysmal growth dynamics.

The rationale for this investigation stems from the emerging recognition that hepatic dysfunction and vascular remodeling share overlapping pathophysiologic mechanisms, including systemic inflammation, oxidative stress, and endothelial injury. Hepatic metabolic stress can alter circulating cytokine profiles, lipid metabolism, and matrix-degrading enzyme activity - all of which have been implicated in aneurysm pathogenesis. Thus, the AST/ALT ratio may serve as a simple, accessible biochemical marker reflecting both hepatic-vascular crosstalk and the systemic inflammatory milieu that drives aneurysm development and expansion.

## METHODS

Our institution conducted a retrospective examination of patients who were diagnosed with AAA between November 2018 and June 2022, with the approval of the local review board (21.05.2024/240085-56). Patients who had been diagnosed with AAAs ranging in diameter from 30 to 54 mm were included in the study. Inclusion criteria for the patient group were a diagnosis of AAA by computed tomography (CT), either incidentally or during evaluation for abdominal pain. The control group consisted of individuals who underwent CT imaging for abdominal pain due to any cause but were found to have no evidence of AAA or hepatobiliary pathology. Controls were retrospectively identified and manually matched to AAA patients in a 1:1 ratio according to age (± 3 years), sex, and major cardiovascular risk factors, including smoking status, hypertension, diabetes mellitus, hyperlipidemia, and family history of coronary artery disease. Propensity score matching was not used because of the limited sample size; however, matching was verified statistically to ensure no significant baseline differences between groups. Patients with a documented history of chronic liver disease, including but not limited to viral hepatitis, autoimmune hepatitis, cirrhosis, and cholestatic liver disorders, evidence of hepatobiliary pathology (*e.g.*, hepatic steatosis, gallstones, or masses) on baseline abdominal imaging (ultrasound or CT), chronic alcohol use as defined by consumption exceeding 20 g/day for women and 30 g/day for men, prior liver biopsy findings consistent with steatohepatitis or fibrosis, current use of hepatotoxic medications or those known to alter liver enzyme levels, such as statins, methotrexate, or antiepileptics, and any prior diagnosis of metabolic liver conditions such as non-alcoholic fatty liver disease or suspected subclinical hepatic steatosis without confirmatory imaging or clinical work-up were excluded.

Baseline and six-month CT scans were analyzed to assess aneurysm growth. In our clinic, the standard protocol for monitoring AAAs larger than 40 mm includes CT evaluations at six-month intervals. Accordingly, follow-up CT images obtained at these intervals were reviewed for each patient. A comprehensive database was used to collect and analyze patient demographics, atherosclerotic risk factors, laboratory findings from hematologic and biochemical tests, and serial aneurysm size measurements from CT imaging.

The study population was divided into two primary groups: the AAA group (n = 90) and the control group (n = 90). The control group consisted of patients who presented to the cardiovascular surgery outpatient clinic with abdominal pain and were matched to the AAA group in terms of age, sex, and cardiovascular risk factors, but had no evidence of AAA on imaging. Patients in the AAA group were further categorized based on aneurysm growth rate determined by serial CT scans obtained six months apart. Rapid aneurysm enlargement was defined as an increase in maximal aortic diameter of ≥ 5 mm within a six-month period, whereas slow enlargement was defined as an increase of < 5 mm over the same interval. This threshold aligns with prior studies identifying a ≥ 5 mm increase as clinically significant for accelerated aneurysmal progression. In total, 180 patients were included in the analysis. The study database was used to assess variables such as age, sex, comorbidities (including diabetes mellitus, smoking, hypertension, hyperlipidaemia, and a family history of coronary artery disease), and laboratory parameters. These included hemogram results, AST and ALT levels, fasting blood glucose, total and direct bilirubin, lipid profile, uric acid, and renal function markers. Additionally, the maximal cross-sectional diameter of the abdominal aorta was recorded from tomographic imaging.

Patients in the AAA group were further classified into two subgroups based on aneurysm progression over a six-month period. Those with an increase in aneurysm diameter of < 5 mm were categorized as the non-rapid expansion group, whereas those with an increase of 5 mm or more were defined as the rapidly expanding aneurysm subgroup.

Blood samples collected in ethylenediaminetetraacetic acid were analyzed using a Sysmex XN1000 haematology analyzer from Sysmex (Kobe, Japan). The analysis included white blood cell (WBC) counts, haemoglobin (Hb) levels, mean cell Hb, red blood cell counts, platelet counts, mean platelet volume, total bilirubin, direct bilirubin, AST, ALT, and glucose level measurements. Low-density lipoprotein (LDL), high-density lipoprotein (HDL), and triglyceride concentrations were determined by enzymatic methods on a COBAS 8000 biochemical analyzer (Roche Diagnostics GmbH; Mannheim, Germany).

Maximal cross-sectional aortic diameter measurements were taken at the initial diagnosis and after six months using cross-sectional views to identify the rapidly growing group. The measurements were recorded in millimetres. The images were acquired through scanning using the same Siemens CT examination (Siemens Definition Flash, Berlin, Germany). All CT evaluations were assessed during the inspiratory phase. The CT evaluation and diameter measurements of the aorta were made by the same researcher.

We calculated the sample size of the study using the G*Power program (v3.1.9.2). We conducted a pilot study with 10 patients for each group. The main study did not use the data from this pilot study. In this pilot study, the mean AST/ALT of patients was 1.32 ± 0.31 in the AAA group and 1.16 ± 0.24 in the control group. Data from 88 patients were required to determine a significant difference with a one-sided type I error (α) of 0.01 and a power (1 - β) of 0.9. We then enrolled 90 patients, compensating for dropouts with the addition of two more.

Descriptive statistics, including means and standard deviations for numerical variables and frequencies and percentages for categorical variables, were provided. Pearson’s chi-square test or Fisher’s exact test and logistic regression were utilized in the analysis of categorical variables. Numerical variables were compared using *t*-tests. R 4.3.2. (R Core Team, 2024) software was employed, and *P* < 0.05 was considered to indicate significance.

Potential collinearity among lipid parameters and multicollinearity diagnostics (variance inflation factors [VIF]) were assessed to confirm that variables were contributing separately to the model. The logistic regression method developed by Firth was employed.

In addition to descriptive and regression analyses, receiver operating characteristic (ROC) curve analysis was performed to evaluate the discriminative performance of the AST/ALT ratio in identifying both the presence of AAA and rapid aneurysm enlargement. The area under the curve (AUC) with corresponding 95% confidence intervals (CI) was calculated to quantify diagnostic accuracy.

The optimal cutoff values were determined using the Youden index. Moreover, the AST/ALT ratio was stratified into quartiles to explore potential dose-response trends and threshold effects in relation to aneurysm risk.

## RESULTS

A total of 180 patients were included in the study, of whom 90 had AAA and 90 were controls. Table 1 presents the demographic and clinical characteristics of patients with AAA compared to matched controls. While age and sex distribution were similar between the groups, hypertension was significantly more common in the AAA group (*P* = 0.036).

**Table 1:** Demographic and clinical characteristics of AAA and control groups.

Variables	AAA	Control	P-value^2^
	n = 90^1^	n = 90^1^	
Sex			0.2
Male	65 (72.22%)	72 (80.00%)	
Female	25 (27.78%)	18 (20.00%)	
Age	65.39 ± 11.44	67.84 ± 11.99	0.2
Smoking	40 (44.44%)	42 (46.67%)	0.8
Hypertension	57 (63.33%)	43 (47.78%)	0.036
Family history of CAD	37 (41.11%)	34 (37.78%)	0.6
Diabetes mellitus	29 (32.22%)	24 (26.67%)	0.4

1 n (%); mean ± standard deviation;

2 Pearson's Chi-squared test; Welch’s two sample *t*-test

AAA=abdominal aortic aneurysm; CAD=coronary artery disease

**Fig. 1:**
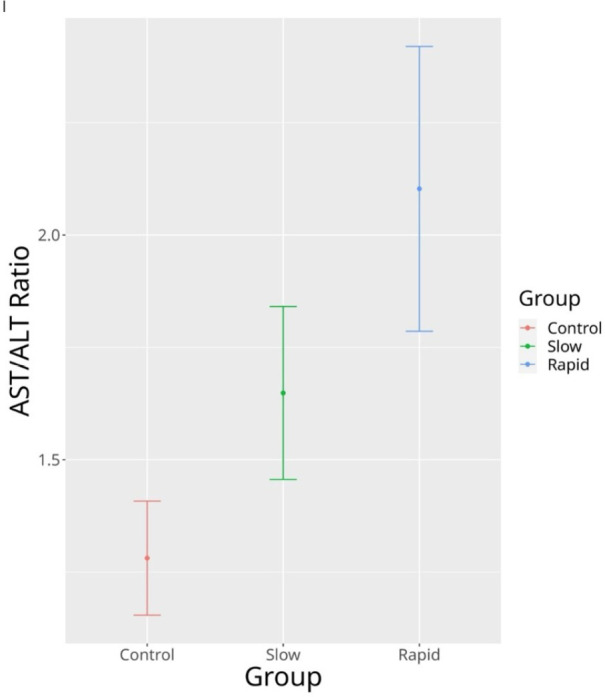
Error bars of the aspartate aminotransferase/alanine aminotransferase (AST/ALT) ratio representing confidence interval.

Table 2 compares the laboratory parameters between the AAA and control groups. The AAA group had significantly higher AST/ALT ratios (1.84 ± 0.84 *vs.* 1.28 ± 0.61; 95% CI: 0.46 - 1.10), triglyceride levels (172.62 ± 67.90 *vs.* 131.70 ± 66.42 mg/dL; 95% CI: 0.31 - 0.91), LDL cholesterol (125.50 ± 33.29 *vs.* 105.21 ± 28.89 mg/dL; 95% CI: 0.35 - 0.95), and WBC counts (9.05 ± 3.51 *vs.* 7.70 ± 2.51 x 10^9^/L ; 95% CI: 0.14 - 0.74). In contrast, HDL cholesterol levels were significantly lower in the AAA group. Figure 1 showed the error bars of the AST/ALT ratio representing CI.

**Table 2:** Laboratory characteristics of AAA and control groups.

Variables	AAA	Control	Difference^2^	95% CI
	n = 90^1^	n = 90^1^		
AST, µ/L	29.92 ± 14.38	21.82 ± 15.46	0.54	0.24, 0.84
ALT, µ/L	18.01 ± 9.14	19.11 ± 11.75	-0.10	-0.40, 0.19
AST/ALT ratio	1.84 ± 0.84	1.28 ± 0.61	0.76	0.46, 1.1
Triglycerides, mg/dL	172.62 ± 67.90	131.70 ± 66.42	0.61	0.31, 0.91
LDL cholesterol, mg/dL	125.50 ± 33.29	105.21 ± 28.89	0.65	0.35, 0.95
HDL cholesterol, mg/dL	45.36 ± 12.11	59.58 ± 15.68	-1.0	-1.3, -0.70
Glucose, mg/dL	117.04 ± 33.20	108.91 ± 33.73	0.24	-0.05, 0.54
Uric acid, mg/dL	4.79 ± 1.32	5.30 ± 2.20	-0.28	-0.58, 0.01
WBC counts × 10^9^/L	9.05 ± 3.51	7.70 ± 2.51	0.44	0.14, 0.74
Haemoglobin, g/dL	13.07 ± 2.35	13.08 ± 2.05	0.00	-0.30, 0.29
MCH, pg	29.05 ± 7.04	29.70 ± 9.34	-0.08	-0.37, 0.21
Red cell distribution width, %	4.84 ± 1.17	4.60 ± 0.87	0.23	-0.06, 0.52
Platelet count, × 10^9^/L	230.90 ± 83.10	239.14 ± 78.64	-0.10	-0.39, 0.19
Mean platelet volume, fL	10.81 ± 1.14	10.67 ± 0.96	0.14	-0.16, 0.43
Neutrophil-lymphocyte ratio	3.43 ± 2.15	3.12 ± 1.40	0.17	-0.12, 0.46
Total bilirubin, µmol/L	1.05 ± 0.32	1.05 ± 0.14	0.01	-0.28, 0.31
Direct bilirubin, µmol/L	0.25 ± 0.03	0.25 ± 0.03	0.01	-0.28, 0.30

1 Mean ± standard deviation;

2 Cohen's D

AAA=abdominal aortic aneurysm; ALT=alanine aminotransferase; AST=aspartate aminotransferase; CI=confidence interval; HDL=high-density lipoprotein; LDL=low-density lipoprotein; MCH=mean cell haemoglobin; WBC=white blood cell

Among patients with AAA, 38 (42%) exhibited rapid aneurysm enlargement, defined as an increase in maximal diameter of ≥ 5 mm over six months, while 52 (58%) showed slow enlargement, defined as < 5 mm increase during the same follow-up period. Table 3 summarizes the baseline demographic and clinical characteristics of patients with slow *vs.* rapid aneurysm enlargement over six months. Although most variables were similar, sex distribution differed significantly between the subgroups (*P* = 0.034).

**Table 3:** Demographic and clinical characteristics of slow and rapid enlargement subgroups.

Variables	Slow Enlargement	Rapid Enlargement	*P*-value^2^	q-value^3^
	n = 52^1^	n = 38^1^		
Sex			0.034	0.2
Male	42.00 (80.77%)	23.00 (60.53%)		
Female	10.00 (19.23%)	15.00 (39.47%)		
Age	65.50 ± 12.05	65.24 ± 10.72	> 0.9	> 0.9
Smoking	23.00 (44.23%)	17.00 (44.74%)	> 0.9	> 0.9
Hypertension	30.00 (57.69%)	27.00 (71.05%)	0.2	0.4
Family history of CAD	22.00 (42.31%)	15.00 (39.47%)	0.8	> 0.9
Diabetes mellitus	14.00 (26.92%)	15.00 (39.47%)	0.2	0.4

1 n (%); mean ± standard deviation;

2 Pearson's Chi-squared test; Welch’s two sample *t*-test;

3 False discovery rate correction for multiple testing

CAD=coronary artery disease

Table 4 highlights the laboratory differences between patients with slow and rapidly expanding AAA. Compared to the slow enlargement group, the rapidly expanding subgroup exhibited significantly higher AST/ALT ratios (2.10 ± 0.96 *vs.* 1.65 ± 0.69; 95% CI: -0.98 to -0.13), triglyceride levels (204.97 ± 87.01 *vs.* 148.98 ± 34.68 mg/dL; 95% CI: -1.30 to -0.46), LDL cholesterol (141.68 ± 36.29 *vs.* 113.67 ± 25.32 mg/dL; 95% CI: -1.40 to -0.48), and WBC counts (10.74 ± 4.06 *vs.* 7.81 ± 2.42 x 10^9^/L ; 95% CI: -1.30 to -0.47). Although Hb levels were also slightly higher in the rapid expansion group (13.64 ± 2.31 *vs.* 12.66 ± 2.31 g/dL), the CI marginally included zero (95% CI: -0.85 to 0.00), indicating borderline significance.

**Table 4:** Laboratory characteristics of slow and rapid enlargement subgroups.

Variables	Slow Enlargement	Rapid Enlargement	Difference^2^	95% CI
	n = 52^1^	n = 38^1^		
AST, µ/L	27.82 ± 13.88	32.79 ± 14.73	-0.35	-0.77, 0.07
ALT, µ/L	18.41 ± 9.32	17.48 ± 8.97	0.10	-0.32, 0.52
AST/ALT ratio	1.65 ± 0.69	2.10 ± 0.96	-0.56	-0.98, -0.13
Triglycerides, mg/dL	148.98 ± 34.68	204.97 ± 87.01	-0.90	-1.3, -0.46
LDL cholesterol, mg/dL	113.67 ± 25.32	141.68 ± 36.29	-0.92	-1.4, -0.48
HDL cholesterol, mg/dL	46.68 ± 13.17	43.55 ± 10.38	0.26	-0.16, 0.68
Glucose, mg/dL	120.18 ± 39.27	112.74 ± 22.17	0.22	-0.20, 0.64
Uric acid, mg/dL	4.86 ± 1.14	4.70 ± 1.54	0.12	-0.30, 0.54
WBC counts × 10^9^/l	7.81 ± 2.42	10.74 ± 4.06	-0.91	-1.3, -0.47
Haemoglobin, g/dl	12.66 ± 2.31	13.64 ± 2.31	-0.42	-0.85, 0.00
MCH, pg	29.09 ± 9.16	28.98 ± 1.86	0.01	-0.40, 0.43
Red cell distribution width, %	4.66 ± 0.89	5.09 ± 1.45	-0.37	-0.79, 0.05
Platelet count, × 10^9^/l	228.27 ± 98.48	234.50 ± 56.74	-0.07	-0.49, 0.34
Mean platelet volume, fL	10.68 ± 0.98	10.99 ± 1.32	-0.27	-0.69, 0.15
Neutrophil-lymphocyte ratio	3.28 ± 2.77	3.65 ± 0.68	-0.17	-0.59, 0.25
Total bilirubin, µmol/l	1.07 ± 0.32	1.02 ± 0.32	0.17	-0.25, 0.59
Direct bilirubin, µmol/l	0.25 ± 0.01	0.25 ± 0.04	-0.12	-0.54, 0.30

1 Mean ± standard deviation;

2 Cohen's D

ALT=alanine aminotransferase; AST=aspartate aminotransferase; CI=confidence interval; HDL=high-density lipoprotein; LDL=low-density lipoprotein; MCH=mean cell haemoglobin; WBC=white blood cell

Table 5 presents the results of a multivariable logistic regression analysis evaluating factors independently associated with the presence of AAA. An elevated AST/ALT ratio was significantly associated with AAA (OR: 2.63; 95% CI: 1.44 - 5.09; *P* = 0.002). Additionally, higher triglyceride levels (OR: 1.01; 95% CI: 1.00 - 1.02; *P* < 0.001) and LDL cholesterol (OR: 1.02; 95% CI: 1.01 - 1.04; *P* < 0.001) were independently associated with AAA, while HDL cholesterol showed a protective effect (OR: 0.93; 95% CI: 0.90 - 0.96; *P* < 0.001), indicating an inverse relationship with aneurysm presence.

**Table 5:** Multivariable logistic regression analysis for AAA (rapid or slow).

Variables	OR	95% CI	*P*-value
AST/ALT ratio	2.63	1.44 - 5.09	0.002
Triglycerides, mg/dL	1.01	1.00 - 1.02	< 0.001
LDL cholesterol, mg/dL	1.02	1.01 - 1.04	< 0.001
HDL cholesterol, mg/dL	0.93	0.90 - 0.96	< 0.001
Hypertension	1.38	0.65 - 2.93	0.4
WBC counts × 10^9^/L	1.00	0.88 - 1.14	> 0.9

AAA=abdominal aortic aneurysm; ALT=alanine aminotransferase; AST=aspartate aminotransferase; CI=confidence interval; HDL=high-density lipoprotein; LDL=low-density lipoprotein; OR=odds ratio; WBC=white blood cell

Table 6 shows the results of a multivariable logistic regression analysis for predictors of rapid aneurysm enlargement. Elevated LDL cholesterol (odds ratio [OR]: 1.04; 95% CI: 1.02 - 1.06; *P* < 0.001), triglyceride levels (OR: 1.01; 95% CI: 1.00 - 1.03; *P* = 0.032), and WBC count (OR: 1.34; 95% CI: 1.09 - 1.73; *P* = 0.009) were significantly associated with rapid aneurysm growth. The AST/ALT ratio was independently associated with the presence of AAA (OR: 2.63; 95% CI: 1.44 - 5.09; *P* = 0.002), though it did not independently predict rapid aneurysmal enlargement (*P* = 0.10).

**Table 6:** Multivariable logistic regression analysis for rapid enlargement.

Variables	OR	95% CI	*P*-value
AST/ALT ratio	1.76	(0.90 - 3.68)	0.10
Triglycerides, mg/dL	1.01	(1.00 - 1.03)	0.032
LDL cholesterol, mg/dL	1.04	(1.02 - 1.06)	< 0.001
WBC counts × 10^9^/L	1.34	(1.09 - 1.73)	0.009

ALT=alanine aminotransferase; AST=aspartate aminotransferase; CI=confidence interval; LDL=low-density lipoprotein; OR=odds ratio; WBC= white blood cell

The VIF values related to Table 5 and Table 6 for AST/ALT ratio, triglycerides, HDL, LDL, and WBC counts were 1.114150, 1.082068, 1.163575, 1.032884, and 1.151840, respectively.

To further assess the discriminative ability of the AST/ALT ratio, ROC curve analyses were performed for both AAA presence and aneurysm progression. For identifying the presence of AAA, the AST/ALT ratio demonstrated good diagnostic performance with an AUC of 0.72 (95% CI: 0.65 - 0.79; *P* < 0.001). For predicting rapid aneurysm enlargement, the discriminative power was moderate, with an AUC of 0.65 (95% CI: 0.54 - 0.76; *P* = 0.002). In quartile-based subgroup analysis, the prevalence of AAA increased progressively across ascending AST/ALT categories (*P* for trend < 0.01). Patients in the highest quartile (AST/ALT ≥ 2.1) had a 3.1-fold higher odds of AAA presence compared with those in the lowest quartile (AST/ALT < 1.2).

**Fig. 2:**
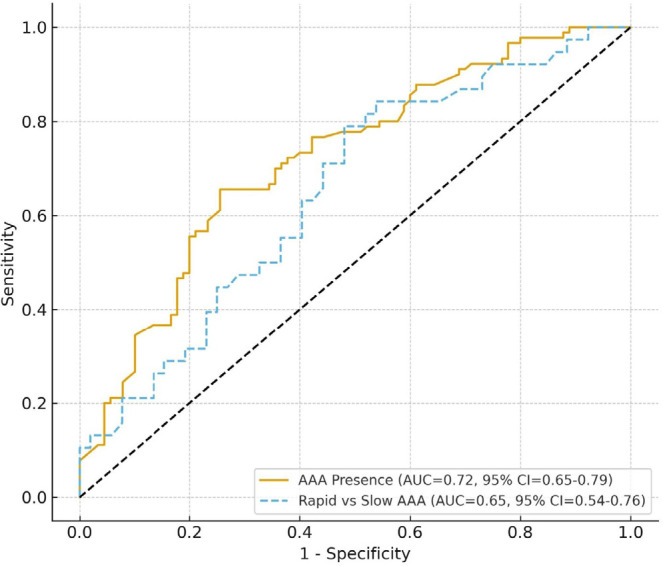
Receiver operating characteristic curves illustrating the discriminative ability of the aspartate aminotransferase/alanine aminotransferase ratio for (A) abdominal aortic aneurysms (AAA) presence and (B) rapid aneurysm enlargement. AUC=area under the curve; CI=confidence interval.

These findings suggest that while the AST/ALT ratio provides clinically relevant discrimination for AAA presence, its predictive utility for rapid aneurysm progression remains modest. The corresponding ROC curves are presented in Figure 2.

## DISCUSSION

This cross-sectional study revealed that an elevated AST/ALT ratio, together with increased levels of triglycerides, LDL cholesterol, and WBC counts, was significantly associated with AAA presence and was higher in patients with rapidly expanding aneurysms, although it did not independently predict aneurysm progression. These associations remained robust after adjusting for various cardiovascular risk factors. Our findings suggest that the AST/ALT ratio - traditionally viewed as a hepatic biomarker - may hold broader systemic relevance, particularly in vascular pathology. Beyond these associations, the ROC curve analysis demonstrated that the AST/ALT ratio has meaningful diagnostic value, showing good discriminative power for AAA presence and moderate ability to predict rapid aneurysm enlargement. Furthermore, quartile-based stratification revealed a stepwise increase in aneurysm prevalence across higher AST/ALT ratio categories, suggesting a potential threshold effect that may reflect systemic inflammatory and metabolic stress.

Recent research supports a model in which Kupffer cell activation in the inflamed liver initiates systemic release of cytokines, particularly interleukin-6 (IL-6), which can act on the vascular wall by engaging the glycoprotein-130 (GP130)/signal transducer and activator of transcription-3 (STAT3) signaling cascade^[7-9]^. Within the aortic media and adventitia, STAT3 activation upregulates matrix metalloproteinases-2 and metalloproteinases-9 (MMP-2, MMP-9) and downregulates tissue inhibitors of metalloproteinases, thereby fostering ECM degradation, elastin fragmentation, and smooth muscle cell phenotypic switching. The IL-6 also promotes endothelial dysfunction, vascular inflammation, and leukocyte recruitment, compounding ongoing vascular remodeling processes. Experimental studies in both animal and *in vitro* models have shown that pharmacologic or genetic inhibition of the IL-6/GP130/STAT3 signaling pathway reduces MMP expression and attenuates aneurysmal dilation, supporting this mechanistic hypothesis^[7,8]^. Moreover, recent cardiovascular and hepatic literature suggests that hepatic inflammatory stress may exert systemic vascular effects even in the absence of overt liver disease^[10-12]^.

In this context, our findings demonstrating that higher AST/ALT ratios (a biochemical surrogate of hepatic inflammatory activity) are associated with both AAA presence and rapid aneurysm progression, providing clinical support for this emerging liver-vascular interaction model. While the association does not establish causality, it underscores the possibility that hepatic inflammation and vascular matrix remodeling are interconnected processes relevant to aneurysm pathophysiology.

Taken together, these findings suggest that the AST/ALT ratio - traditionally regarded as a hepatic biomarker - may also serve as a readily available surrogate marker for vascular dysfunction and aneurysmal disease risk.

Several studies have previously linked elevated AST/ALT ratios with poor outcomes in cardiovascular conditions such as coronary artery disease and heart failure. Liu et al. demonstrated that a higher AST/ALT ratio was associated with all-cause mortality in patients with stable coronary artery disease^[4]^. Likewise, Bezan et al. reported its prognostic value in renal cancer patients, underscoring its cross-systemic relevance^[13]^. Notably, in a Chinese hypertensive population, Liu et al. found a significant association between AST/ALT ratio and peripheral artery disease, supporting its role as a vascular biomarker^[3]^. Khan et al. also demonstrated that elevated AST levels correlate with increased MMP activity and ECM degradation - key processes in aneurysm formation and progression^[14]^. These findings imply that the AST/ALT ratio may serve not only as a surrogate of liver dysfunction or metabolic syndrome but also as a marker of systemic vascular remodeling. Therefore, its association with AAA progression likely reflects a multifactorial interplay between systemic inflammation, liver-derived cytokines, and vascular wall instability. These findings complement our own results, particularly regarding the elevated AST/ALT ratio and WBC count seen in patients with rapidly expanding aneurysms. Our data may reflect these same underlying mechanisms, where systemic inflammation and vascular matrix degradation lead to structural weakening of the aortic wall. The elevated AST/ALT ratio may serve as an indirect marker of these pathological processes, supporting its potential use in AAA risk stratification.

In line with these observations, our study proposes that elevated AST/ALT levels may not merely indicate liver dysfunction but also reflect a pro-inflammatory or pro-atherogenic state conducive to aneurysmal development. Moreover, it is known that reduced hepatic blood flow - often occurring in subclinical cardiac dysfunction - can lead to disproportionate elevations in AST relative to ALT. Yokoyama et al. showed that such shifts in transaminase ratios were associated with elevated brain natriuretic peptide levels and cardiovascular mortality in the general population^[15]^.

Consistent with this hepatic-vascular interaction, our results also showed decreased ALT values in patients with rapidly expanding aneurysms, contributing to a higher AST/ALT ratio. This phenomenon may represent cardio-hepatic crosstalk, where vascular stiffness, inflammation, or ischemia impairs hepatic perfusion, influencing enzyme release patterns. The concept of cardio-hepatic syndrome reinforces the interconnectedness of these two organ systems and supports a systemic approach in risk evaluation^[16,17]^.

The association observed in our study between an elevated AST/ALT ratio and adverse cardiovascular outcomes aligns with the findings of Nakashima et al., who demonstrated the prognostic utility of the Fibrosis-5 (FIB-5) index in patients with severe isolated tricuspid regurgitation^[18]^. Since FIB-5 incorporates AST, ALT, albumin, alkaline phosphatase, and platelet count, their results highlight the broader relevance of liver function markers in cardiovascular risk stratification. While our study specifically focused on the AST/ALT ratio, the consistency with FIB-5-based risk prediction supports the concept that subclinical hepatic dysfunction may contribute to adverse cardiac remodeling and systemic inflammation. These parallels underscore the emerging role of liver-derived biomarkers in cardiovascular prognostication and suggest that simple, readily available indices such as the AST/ALT ratio could serve as practical tools in the clinical evaluation of patients at risk for aortic pathology.

Beyond transaminase ratios, we observed that dyslipidaemia - particularly high triglyceride and LDL levels - was significantly associated with AAA presence and growth. This supports previous work by Zheng et al., who highlighted shared genetic susceptibility between AAA and lipid metabolism pathways^[1]^.

Furthermore, recent experimental studies offer mechanistic insight into how liver-derived factors may contribute to aneurysm biology. Wang et al. showed that quercetin, a flavonoid with hepatoprotective and anti-inflammatory properties, significantly reduced AST and ALT levels while suppressing AAA progression in murine models^[19]^. Zhao et al. also reported the hepatoprotective potential of quercetin across various models of liver injury^[20]^.

Recent research in cardiovascular surgery has emphasized the clinical relevance of liver function parameters in postoperative outcomes. For instance, a study by Baysal et al. investigated hyperbilirubinemia following open-heart surgery and found that prolonged cardiopulmonary bypass time, low ejection fraction, and extended intensive care unit stay were significant predictors of postoperative hepatic dysfunction^[11]^. These findings reinforce the concept that liver-derived biochemical markers may reflect not only hepatic but also systemic circulatory stress, supporting our interpretation of elevated AST/ALT ratios as a manifestation of systemic metabolic disturbance rather than isolated hepatocellular injury. In light of these perspectives, the AST/ALT ratio emerges as a promising adjunct marker reflecting underlying vascular stress and low-grade inflammation, particularly in patients at risk for aneurysmal disease. While our study focuses specifically on AAA, this broader pathophysiologic relevance suggests that simple laboratory tests like the AST/ALT ratio might be incorporated into routine preoperative evaluation to help refine clinical decision-making and follow-up strategies.

Importantly, our data also confirm the association between elevated WBC counts and AAA, especially in rapidly growing cases. This aligns with studies by Iribarren et al. and Vuruşkan et al., who identified leukocyte activation as a potential driver of aneurysm expansion. Inflammatory markers such as the C-reactive protein/albumin ratio and neutrophil-to-lymphocyte ratio may also complement traditional lipid and hepatic profiles in future risk stratification models^[21,22]^. Importantly, VIF values for the AST/ALT ratio, triglycerides, HDL, LDL, and WBC were all < 1.2, suggesting no significant multicollinearity among these variables. This supports the validity of the regression analysis, reinforcing our finding that the AST/ALT ratio is independently associated with the presence of AAA, and that lipid and inflammatory markers are robust predictors of aneurysmal growth.

This study possesses several notable strengths. First, it explores a novel association between the AST/ALT ratio and AAA progression, addressing a gap in the current literature. Second, the study includes a relatively large and well-matched patient cohort with detailed clinical, laboratory, and radiological data, allowing for comprehensive statistical analysis and subgroup evaluation. Third, the exclusion of patients with overt hepatic disease and the control for common cardiovascular risk factors enhance the internal validity of the findings. Lastly, the use of objective imaging criteria based on computed tomography at baseline and follow-up strengthens the reliability of aneurysm size measurements and progression assessment.

### Limitations

It is important to note that this study demonstrates association rather than causation. Given its cross-sectional design, no direct causal relationship between elevated AST/ALT ratios and AAA development or progression can be inferred. The AST/ALT ratio may be influenced by systemic inflammatory or metabolic conditions that elevate transaminase levels independent of aneurysmal processes. Therefore, while the observed associations suggest that hepatic-vascular crosstalk and inflammation play a role in AAA pathophysiology, they do not establish a direct mechanistic link. Prospective longitudinal studies and mechanistic investigations are warranted to clarify whether changes in hepatic enzyme patterns contribute to aneurysm initiation or merely reflect underlying systemic inflammation.

This study has several limitations that should be acknowledged. First, its single-centre and retrospective design may limit the generalizability of the findings to broader populations. Second, the AST/ALT ratio was measured at only one time point, which prevents evaluation of longitudinal changes or temporal associations. Third, although patients with overt liver disease were excluded, subclinical hepatic conditions could not be fully ruled out, as advanced diagnostic tools such as elastography were not utilized. Finally, potential confounding factors - including medication use, dietary habits, and lifestyle variables - were not comprehensively controlled, which may have influenced the observed associations.

## CONCLUSION

In summary, this study demonstrated that an elevated AST/ALT ratio is significantly associated with the presence of AAA, reflecting possible hepatic-vascular inflammatory crosstalk. However, the ratio did not independently predict aneurysm progression, indicating that its value lies in association rather than prognostication. From a clinical standpoint, the AST/ALT ratio may serve as a readily accessible biochemical marker that complements traditional cardiovascular risk assessments, but it should not be interpreted as a stand-alone determinant for surveillance or management decisions. Future longitudinal and mechanistic studies are warranted to clarify whether hepatic inflammatory activity contributes causally to aneurysm development or simply mirrors systemic inflammatory processes involved in vascular remodeling.

## Data Availability

The data underpinning the findings of this investigation can be acquired from the corresponding author upon a reasonable request. Individual participant data cannot be publicly released owing to privacy and ethical considerations. The aggregated data and summary statistics used in the analysis are available and can be supplied to qualified researchers upon request.

## Artificial Intelligence Usage

The authors declare that no artificial intelligence tool was used in the preparation of this article.
